# IF-combined smRNA FISH reveals interaction of MCPIP1 protein with *IER3* mRNA

**DOI:** 10.1242/bio.018010

**Published:** 2016-06-02

**Authors:** Jakub Kochan, Mateusz Wawro, Aneta Kasza

**Affiliations:** Department of Cell Biochemistry, Faculty of Biochemistry, Biophysics and Biotechnology, Jagiellonian University, Cracow 30-387, Poland

**Keywords:** IER3, MCPIP1, Transcripts turnover, Inflammation

## Abstract

MCPIP1 and IER3 are recently described proteins essential for maintenance of immune homeostasis. IER3 is involved in the regulation of apoptosis and differentiation and has been shown lately to protect activated T cells and macrophages from apoptosis. MCPIP1 is an RNase critical for controlling inflammation-related mRNAs. MCPIP1 interacts with and degrades a set of stem-loop-containing mRNAs (including *IL-6*). Our results demonstrate the involvement of MCPIP1 in the regulation of *IER3* mRNA levels. A dual luciferase assay revealed that over-expression of MCPIP1 resulted in a decrease of luciferase activity in the samples co-transfected with constructs containing luciferase CDS attached to *IER3* 3′UTR. We identified a stem-loop structure similar to that described to be important for destabilization of the *IL-6* mRNA by MCPIP1. Examination of *IER3* 3′UTR sequence, structure and evolutionary conservation revealed that the identified stem-loop is buried within a bigger element. Deletion of this fragment abolished the regulation of *IER3* 3′UTR-containing transcript by MCPIP1. Finally, using immunofluorescence-combined single-molecule RNA FISH we have shown that the MCPIP1 protein co-localizes with *IER3* mRNA. By this method we also proved that the presence of the wild-type NYN/PIN-like domain of MCPIP1 correlated with the decreased level of *IER3* mRNA. RNA immunoprecipitation further confirmed the interaction of MCPIP1 with *IER3* transcripts *in vivo*.

## Abstract (precis)

**Summary:** We identify *IER3* mRNA as a newly discovered MCPIP1 target using recently described IF-based procedures, and also identify a conserved element involved in MCPIP1-dependent *IER3* transcript destabilization.

## INTRODUCTION

Inflammation is a crucial component of the immune response that allows organisms to deal with the loss of cellular and tissue homeostasis, repair tissue damage and fight invading pathogens. Inflammatory reaction is, however, a double-edged sword. Exaggerated response may lead to severe tissue damage. For a rapid induction and efficient resolution of the inflammation, gene expression in cells of the immune system is tightly regulated at the transcriptional and post-transcriptional level (Anderson, 2008, 2009, 2010; Hao and Baltimore, 2009; Medzhitov and Horng, 2009).

*Immediate early response gene 3* (*IER3*) belongs to the group of genes rapidly activated during inflammation. Its expression is induced by pro-inflammatory cytokines such as TNF and IL-1β and by products of bacterial and viral infections (Arlt and Schäfer, 2011; Kochan et al., 2014; Kondratyev et al., 1996). IER3 is involved in the regulation of cell apoptosis. It exerts pro-apoptotic effect in cells growing in unfavorable growth conditions through interference with the ubiquitin-proteasome-pathway (Arlt and Schäfer, 2011). The importance of IER3 in the proper course of inflammation was confirmed in studies with the use of transgenic mice. Mice overexpressing IER3 in T cells exhibit higher susceptibility to a lupus-like autoimmune disease due to the impaired apoptosis of activated T cells and thus extended duration of effector-phase of immune response (Zhang et al., 2002). Similarly, bone-marrow derived macrophages from *Ier3* knockout mice showed reduced survival upon activation, indicating that Ier3 protects macrophages from LPS-induced cell death (Schott et al., 2014).

For many years the studies of gene expression were focused on the control of transcriptional regulation; however promoter activation is just one of many stages in the processes leading to the final synthesis of protein. Large-scale transcriptome analyses indicate that as many as half of all changes in the amount of transcript may result from altered rates of mRNA decay. Transcripts turnover is regulated by proteins and miRNAs interacting with *cis*-acting elements within the mRNA. These *cis*-acting determinants can be found in untranslated regions (UTRs) or the coding regions of the transcripts. Among conserved elements present in 3′UTRs of the majority of inflammation- and cancer-associated transcripts are the adenylate-uridylate-rich sequences (AREs). One of the best-characterized ARE-binding zinc finger protein is tristetraprolin (TTP) (Blackshear, 2002); binding of TTP to ARE increases the rate of mRNA deadenylation by recruiting the multisubunit Ccr4-Caf1-Not deadenylase complex, thus accelerating its degradation (Fabian et al., 2013). TTP targets belong to the ‘immediate-early response’ genes exhibiting rapid induction and subsequent rapid decay kinetics, one of which is *IER3* mRNA (Lai et al., 2006). ARE elements present in *IER3* 3′UTR are not only involved in the transcript turnover but also mediate its translational regulation upon LPS stimulation. In resting mouse macrophages translation of *Ier3* mRNA is strongly suppressed in an ARE-dependent manner resulting in barely detectable synthesis of protein, independent of high mRNA level. Upon LPS treatment translation is de-repressed and the protein is synthesized (Schott et al., 2014). During macrophage stimulation ARE-mediated mRNA decay is transiently inhibited by upstream signaling events. In contrast other elements, such as constitutive decay element (CDE), are involved in the constitutive mRNA degradation (Stoecklin et al., 2003). CDE is a conserved RNA stem-loop motif that is recognized by Roquin. Indeed *IER3* mRNA 3′UTR contains CDE and is a Roquin target (Leppek et al., 2013). Similarly to TTP, Roquin interacts with the Ccr4-Caf1-Not deadenylase complex leading to increased degradation of *IER3* transcript (Leppek et al., 2013). Recently it has been published that Roquin controls translationally inactive mRNAs and that an overlapping set of these mRNAs is also recognized by an ARE-independent RNase, MCPIP1 (also known as ZC3H12A or Regnase-1) (Mino et al., 2015). MCPIP1 associates with mRNAs encoding inflammation-related proteins, such as IL-6, IL-1β, IL-12p40, IL-2 and c-Rel, accelerating their degradation (Kochan et al., 2015; Li et al., 2012; Matsushita et al., 2009; Mizgalska et al., 2009). In contrast to Roquin, MCPIP1 specifically cleaves and degrades translationally active mRNAs and requires the helicase activity of UPF1 (Mino et al., 2015). Similarly to IER3, MCPIP1 is also involved in the regulation of the proper course of inflammation although the mechanism is completely different. The importance of MCPIP1 in the development of inflammation was proved by the use of knock-out mice; *Zc3h12a−/−* mice (lacking functional gene coding for MCPIP1 protein) spontaneously develop severe autoimmune inflammatory disease and most of them die within 12 weeks of birth (Matsushita et al., 2009). The regulation of *IER3* mRNA turnover by MCPIP1 was not investigated until now.

In this study we present the *IER3* mRNA to be a novel MCPIP1 target. We identified a conserved element containing a stem-loop structure involved in the destabilization of this transcript by MCPIP1. Using immunofluorescence (IF)-combined single-molecule RNA FISH (smRNA FISH), a procedure we developed and described previously in Kochan et al. (2015), we have also supplied evidence for the interaction of MCPIP1 protein with *IER3* mRNA *in vivo* and for the importance of a MCPIP1 NYN/PIN-like domain in the destabilization of endogenous *IER3* transcript. The interaction of *IER3* mRNA with MCPIP1 *in vivo* was further supported by the use of RNA immunoprecipitation (RIP).

## RESULTS

### IER3 3′UTR contains conserved elements potentially recognized by MCPIP1

**Fig. 1.**
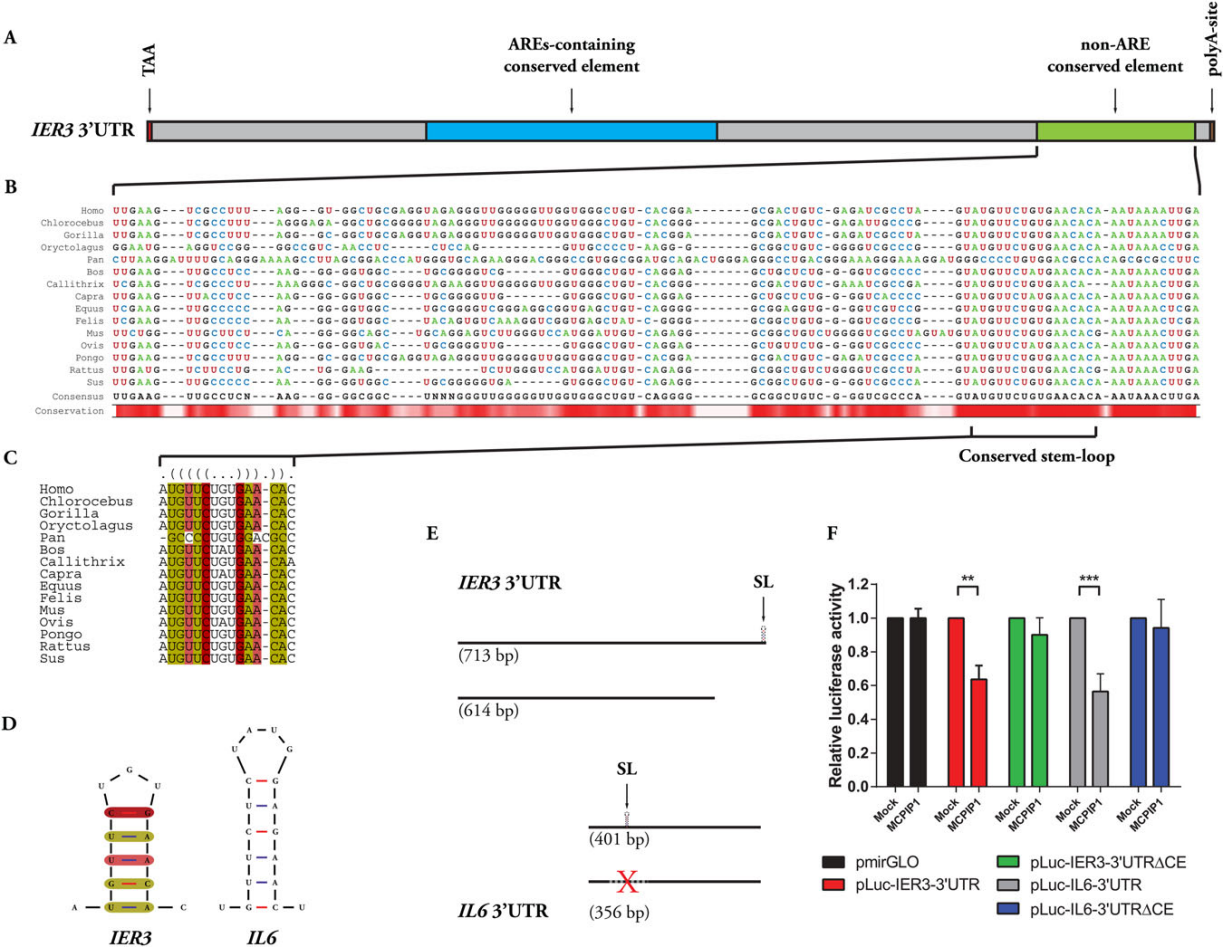
**MCPIP1 regulates *IER3* mRNA stability.** (A) Diagram depicting human *IER3* 3′UTR. Elements described in the text are highlighted and presented according to scale. (B,C) Clustal Omega sequence alignment (B) of *IER3* 3′UTR non-ARE conserved element of 15 various mammalian species revealing presence of a structurally conserved stem-loop further analyzed using LocARNA web server (C). (D) Visual comparison of stem-loops found in 3′UTRs of *IER3* and *IL6* (folded using mfold software with default settings; structures with lowest ΔG are presented). Compatible base pairs are colored. The saturation decreases with the number of incompatible base pairs indicating the structural conservation of the base pair. Color legend in (C) and (D) corresponds to each other. (E-F) Luciferase reporter gene assay results proving the identification of *IER3* 3′UTR element conferring responsiveness to MCPIP1. HepG2 cells were co-transfected with pLUC-IER3-3′UTR, pLuc-IER3-3′UTRΔCE, pLUC-IL-6-3′UTR, pLuc-IL-6-3′UTRΔCE (constructs schematically depicted in E) or empty pmirGLO and MCPIP1-MycHis expression vector. Red X indicates deletion of conserved element recognized by MCPIP1. Graph shows mean±s.d. results from three independent experiments; ***P=0.0002, **P=0.0038. Applied statistical method: two-way ANOVA with Bonferroni *post hoc* test.

We demonstrated previously that transcription factor Elk-1 is involved in IL-1β-dependent regulation of *ZC3H12A* (coding for MCPIP1) and *IER3* expression (Kasza et al., 2010; Kochan et al., 2014). Our results indicated also the existence of a negative regulatory loop aimed at controlling the synthesis of IL-1β. IL-1β induces the expression of MCPIP1, an RNase that plays a crucial role in the degradation of *IL-1β* mRNA (Kasza et al., 2010; Mizgalska et al., 2009). Seeing that both IER3 and MCPIP1 share a common regulatory factor, and taking into account that MCPIP1 is essential for destabilization of mRNAs playing a key role in the inflammatory response and immune homeostasis, we decided to examine the possibility of MCPIP1-mediated control of *IER3* mRNA. We searched the *IER3* 3′UTR for *cis*-acting elements that may be required for MCPIP1-driven mRNA destabilization; Clustal Omega (Li et al., 2015) sequence alignment of *IER3* 3′UTRs of fifteen various mammalian species revealed the presence of two highly conserved elements (Fig. 1A). Detailed analysis of these regions indicated the first one to be a highly conserved AU-rich region containing all AREs described formerly as responsible for TTP-dependent destabilization of *IER3* mRNA (Lai et al., 2006) and further supported by ARE prediction using AREsite2 database (Fallmann et al., 2015). The second element, however, did not show any AU-rich properties; since MCPIP1 is an ARE-independent regulatory RNase (Matsushita et al., 2009), we decided to analyse the non-ARE conserved element (Fig. 1B) deeper. Using mfold software (Zuker, 2003), we identified a stem-loop structure embedded in the non-ARE conserved element of human *IER3* 3′UTR that resembled one important for *IL-6* mRNA destabilization (Paschoud et al., 2006) and was later described as an element recognized by MCPIP1 (Matsushita et al., 2009) (Fig. 1D). With this in hand we decided to verify the structural conservation of the identified stem-loop. Using the LocARNA web server (Smith et al., 2010; Will et al., 2007, 2012) we managed to compare the same set of mammalian species *IER3* 3′UTRs and found an excellent structural conservation of this element, which reached almost 100% (Fig. 1C).

### Involvement of the identified conserved element in MCPIP1-dependent transcript degradation

We used a reporter gene system based on constructs containing the luciferase coding sequence (CDS) with attached 3′UTR of *IER3* mRNA (pLuc-IER3-3′UTR) to verify the hypothesis that *IER3* is an MCPIP1 target. Overexpression of MCPIP1 resulted in a decrease of luciferase activity in the samples co-transfected with construct containing investigated 3′UTR, suggesting that the addition of *IER3* 3′UTR confers responsiveness to MCPIP1 (Fig. 1F). Next, we decided to apply the strategy used in the report analysing MCPIP1-responsive regions in the IL-6 3′UTR (Matsushita et al., 2009). We attached to the luciferase CDS a truncated variant of *IER3* 3′UTR (without non-ARE conserved element containing structurally conserved stem-loop) (Fig. 1E). Deletion of the conserved fragment abolished the regulation of *IER3* 3′UTR-containing transcript by MCPIP1 (Fig. 1F). Similarly, overexpressed MCPIP1 had no effect on luciferase activity in samples co-transfected with a plasmid containing the luciferase CDS with the *IL-6* 3′UTR with deleted conserved element containing specific stem-loop structure (pLuc-IL6–3′UTRΔCE) (Fig. 1E,F). MCPIP1 did not alter luciferase activity in samples co-transfected with control plasmid containing the luciferase CDS alone, with no 3′UTR added (pmirGLO) (Fig. 1F).

### Establishment of smRNA FISH setup for IER3 mRNA quantification

**Fig. 2.**
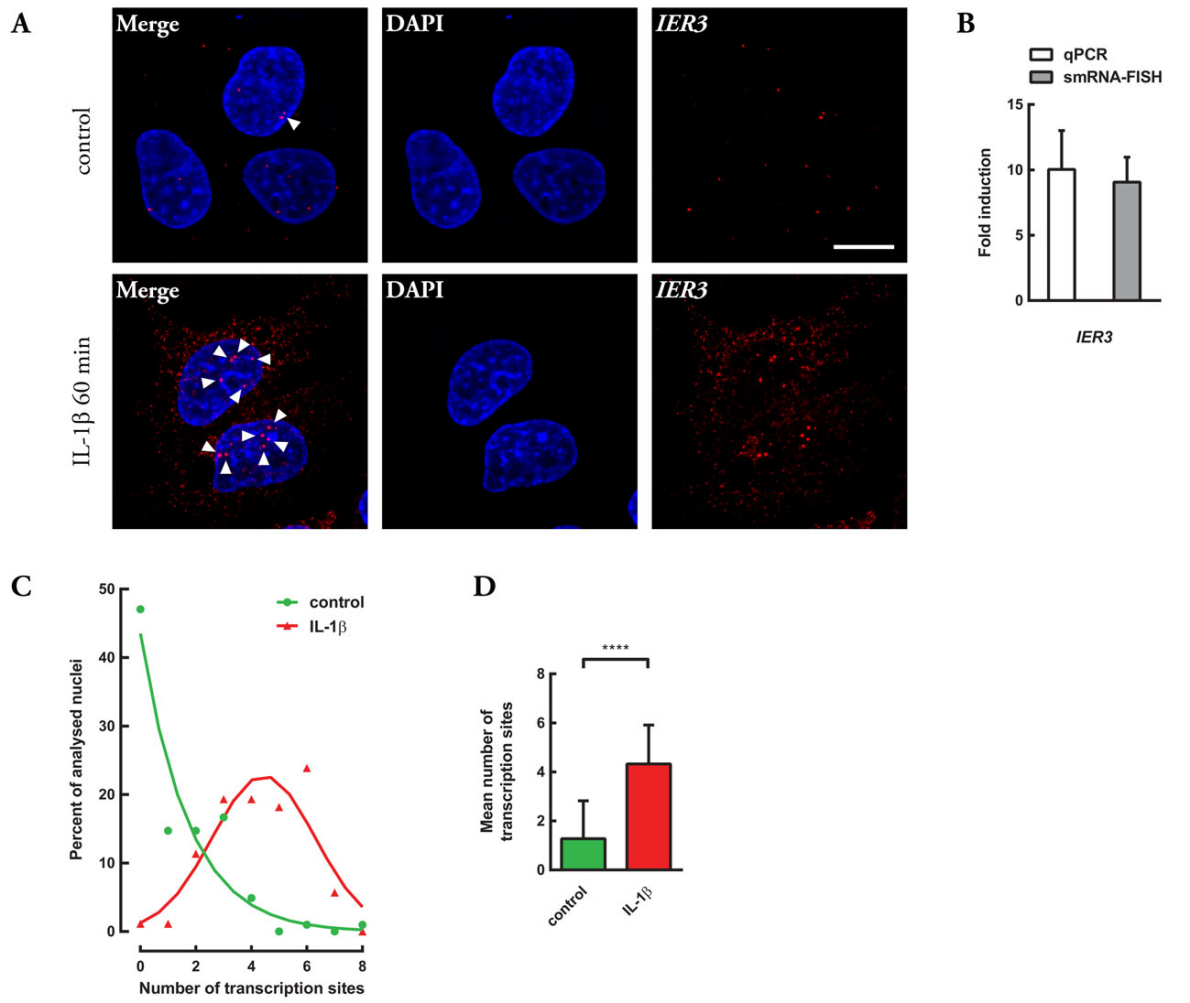
**Establishment of smRNA FISH setup for *IER3* mRNA quantification.** (A) Representative fluorescence images of HepG2 cells stimulated with interleukin 1β (IL-1β) for 60 min and control cells (DAPI, nuclei; *IER3*, probe blend labeled with fluorescent dye Quasar 570). (B) Comparison of results obtained by the use of smRNA FISH and quantitative real-time PCR (qPCR). (C,D) Quantitative analysis of *IER3* active transcription sites observed in HepG2 cells using smRNA FISH. (C) Active transcription site frequency distribution in control and IL-1β-stimulated cells. The Gaussian curves were created by nonlinear regression of the frequency distribution. (D) Graph presenting mean number of active transcription sites in control and IL-1β stimulated cells. Applied statistical method: *t*-test, two-tailed. (B) qPCR: *n=*3, smRNA FISH: fold induction calculated from *n=*51 for control and *n=*64 for IL-1β stimulated cells. (C,D) *n=*102 for control and *n=*88 for IL-1β stimulated cells. Means±s.d., ****P<0.0001. White arrowheads in A indicate active transcription sites. Scale bar: 10 μm.

Having verified the regulation of the transcript containing *IER3* 3′UTR by MCPIP1 using the reporter gene system, we decided to examine whether MCPIP1 interacts *in vivo* with endogenous *IER3* mRNA. To address this issue we took advantage of the method enabling simultaneous protein and mRNA detection using immunofluorescence (IF) and single-molecule RNA fluorescence *in situ* hybridization (smRNA FISH) (Kochan et al., 2015). We used a custom-made set of Stellaris probes targeting the whole *IER3* transcript. Guided by our experience with this procedure, we decided first to ascertain that the designed probe blend produced a specific and quantitative signal. It was shown previously that smRNA FISH results may correspond well to qRT-PCR data (Kochan et al., 2015; Raj et al., 2008); therefore we decided to verify whether the signal observed in smRNA FISH targeting *IER3* is comparable to the qRT-PCR results. We stimulated HepG2 cells with IL-1β, a cytokine known to rapidly and highly induce *IER3* mRNA (Arlt and Schäfer, 2011; Kochan et al., 2014), and subsequently performed both smRNA FISH and qRT-PCR analyses. In line with our expectations, smRNA FISH revealed strong induction of *IER3* mRNA after IL-1β treatment as measured by the number of *IER3* mRNA particles per cell (Fig. 2A). Detailed quantification and comparison of obtained results provided evidence that smRNA FISH counts corresponded well to qRT-PCR data of the fold induction (Fig. 2B) demonstrating that the smRNA FISH signal comes from *IER3* transcripts and not other mRNAs, and that our procedure allowed for detection of the vast majority of, if not all, *IER3* mRNA molecules in the imaged specimens. It is worth noticing that the fluorescent images also clearly showed sites of actively transcribed regions in the genome i.e. large clusters of nascent mRNAs (visible as bright spots in nuclei, marked by white arrowheads in Fig. 2A). Quantitative analysis of active transcription sites frequency distribution in control and IL-1β stimulated HepG2 cells revealed a robust increase in their number and large shift in the distribution towards higher counts (larger number of observed active transcription sites per cell) upon IL-1β treatment (Fig. 2C,D). The first striking observation of more than two actively-transcribed regions in the genome of human cells expected to be diploid finds support in the literature. HepG2 cells have been reported to show prominent amplification/segmental gains involving chromosome 6, especially at 6p21.1-p25.2, a chromosome region where *IER3* gene is located (Kwon et al., 2013; López-Terrada et al., 2009). These results provide another line of evidence corroborating the specificity of the *IER3* smRNA FISH probe blend, thus, the data presented above demonstrated the effectiveness of our smRNA FISH setup.

### MCPIP1 protein co-localizes with IER3 mRNA

**Fig. 3.**
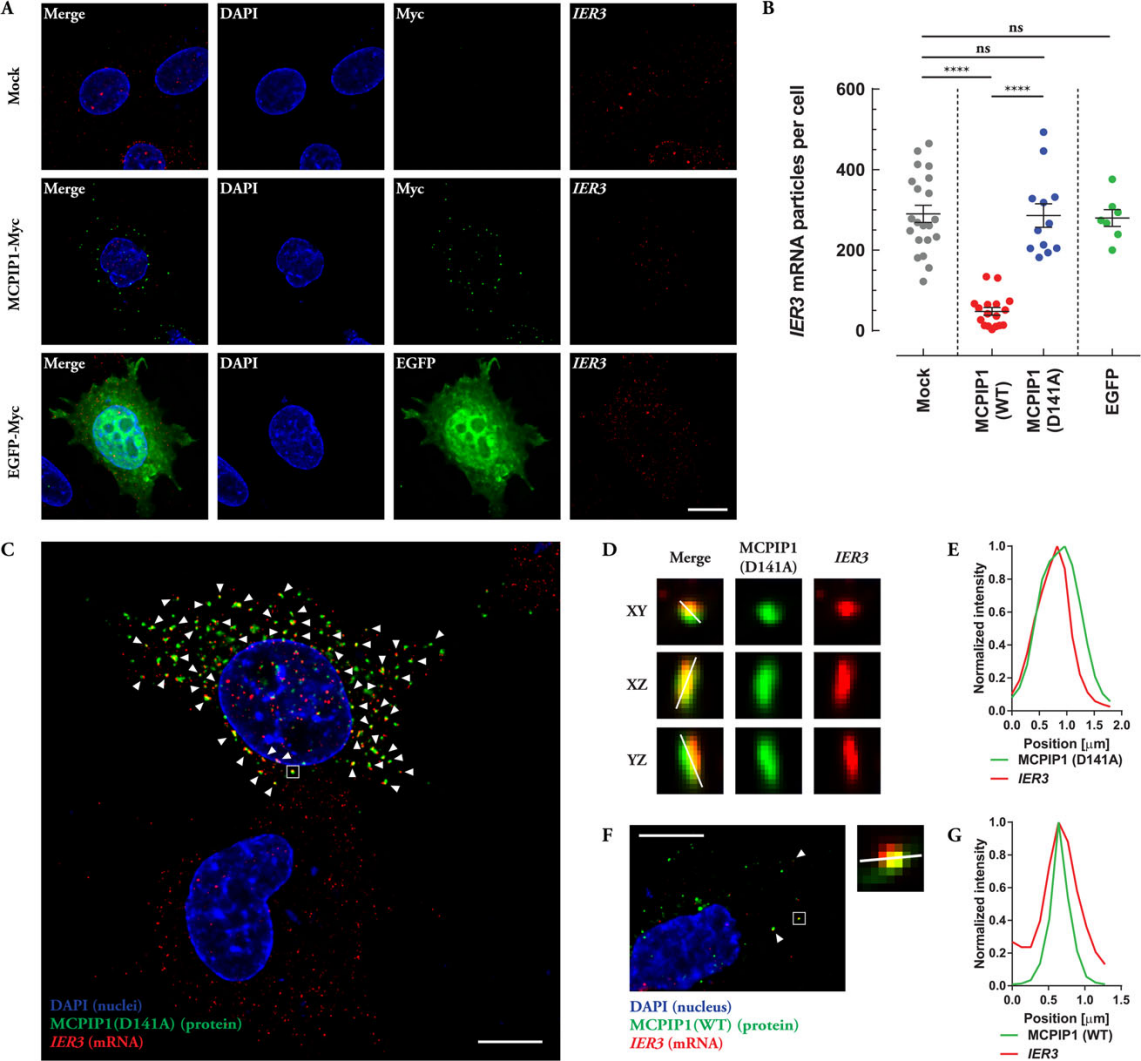
**IF-combined smRNA FISH analysis of MCPIP1 protein interaction with *IER3* mRNA.** (A) Representative fluorescence images of HepG2 cells transfected with empty vector (mock, upper panel) or with expression vectors coding for MCPIP1-MycHis (middle panel) or EGFP-MycHis (lower panel) and subsequently subjected to IF-combined smRNA FISH procedure (DAPI, nuclei; Myc, primary antibody: mouse anti-c-myc, secondary antibody: Alexa Fluor 488-conjugated donkey anti-mouse; EGFP, native fluorescence of EGFP-MycHis; *IER3*, probe blend labeled with fluorescent dye Quasar 570). (B) Quantitative analysis of IF-combined smRNA FISH images of HepG2 cells transfected with empty vector (mock) or with expression vectors coding for MCPIP1-MycHis, MCPIP1(D141A)-MycHis or EGFP-MycHis. Column scatter graph presents all analyzed cells with mean number of *IER3* mRNA particle count for each experimental variant. Error bars=s.e.m. Applied statistical method: one-way ANOVA with Bonferroni *post hoc* test. Numbers of analyzed cells: mock, *n=*21, MCPIP1-MycHis, *n=*17; MCPIP1(D141A)-MycHis, *n=*12 and EGFP-MycHis, *n=*7. ns, not significant; ****P<0.0001. (C) Representative IF-combined smRNA FISH image of HepG2 cells transfected with expression vector coding for MCPIP1(D141A)-MycHis (DAPI, nuclei; MCPIP1(D141A) (protein), primary antibody: mouse anti-c-myc, secondary antibody: Alexa Fluor 488-conjugated donkey anti-mouse; *IER3* (mRNA), probe blend labeled with fluorescent dye Quasar 570). White arrowheads indicate co-localization of MCPIP1 with *IER3* mRNA. Boxed area is presented in more detail in (D). (E) An intensity line profile illustrating the MCPIP1/*IER3* co-localization observed in D. (F) Representative IF-combined smRNA FISH image of HepG2 cells transfected with expression vector coding for MCPIP1(WT)-MycHis (DAPI, nuclei; MCPIP1(WT) (protein), primary antibody: mouse anti-c-myc, secondary antibody: Alexa Fluor 488-conjugated donkey anti-mouse; *IER3* (mRNA), probe blend labeled with fluorescent dye Quasar 570). White arrowheads indicate co-localization of MCPIP1 with *IER3* mRNA. Boxed area is presented in more detail next to the main image (*xy* plane projection). (G) An intensity line profile illustrating the MCPIP1/*IER3* co-localization observed in F. Scale bars: 10 μm in A,C,F (main); 1.83 μm in D; 1.32 μm in F (zoom). Co-localization was assessed using Huygens Professional Co-localization Analyzer (Scientific Volume Imaging, SVI, Hilversum, The Netherlands).

Finally, after demonstrating functionality of the designed smRNA FISH setup, we could start experiments leading to examine interaction of MCPIP1 protein with endogenous *IER3* mRNA. It was also interesting to see if ectopically expressed MCPIP1 influences the level of *IER3* transcript. In order to test this speculation, we transiently transfected HepG2 cells with an expression vector coding for MCPIP1-MycHis or an RNase-inactive mutant of MCPIP1, MCPIP1(D141A)-MycHis. Cells transfected with an empty (pcDNA3.1/MycHisA) or an EGFP-MycHis expression vector served as negative controls in these experiments. Following transfection, IF-combined smRNA FISH procedure was performed as described previously (Kochan et al., 2015). Wild-type (WT) and mutant (D141A) lacking RNase activity version of MCPIP1 proteins were detected using anti-c-myc antibodies; EGFP was detected using its own native fluorescence and *IER3* mRNA was detected using the smRNA FISH probe blend tested above (Fig. 3). We observed significant decrease in *IER3* mRNA level in cells transfected with wild-type MCPIP1 (Fig. 3A,B). Quantitative analysis of imaged cells revealed lower counts of *IER3* mRNA particles in those cells (about one third of control or less) while cells transfected with MCPIP1(D141A), EGFP or mock-transfected did not show any alterations in *IER3* mRNA level (Fig. 3B). These results clearly show that MCPIP1 is involved in the control of endogenous *IER3* mRNA turnover and that this regulation is Asp141-dependent, pointing to the RNase activity of MCPIP1 to be crucial in the observed regulation. Eventually we were able to see, for the first time, co-localization of the MCPIP1 protein with the *IER3* transcript. Pronounced and clearly visible co-localization was observed when a mutant form of MCPIP1 protein (D141A) (lacking RNase activity but with intact RNA binding properties) was used (Fig. 3C-E). By taking advantage of the fact that transient transfection is far from being 100% effective we could image successfully transfected cells surrounded by cells without ectopically expressed protein in the same field of view. Fig. 3C transparently depicts such situation. This image not only shows immense co-localization of MCPIP1(D141A) with endogenous *IER3* mRNA (nearly all fluorescent spots originating from anti-c-myc staining overlay with *IER3* particles within this cell, indicated by white arrowheads) but also allows for direct comparison of the number of *IER3* mRNA spots in a transfected and a control cell proving no alteration in *IER3* mRNA level in a cell transfected with RNase-inactive form of MCPIP1. The co-localization is also observed when wild-type MCPIP1 was overexpressed in HepG2 cells (Fig. 3F,G, white arrowheads and box). As MCPIP1 is a nuclease it may seem to be conceptually unexpected to observe co-localization with its substrate mRNAs since the RNase activity should be quickly followed by target degradation rendering MCPIP1 bound mRNAs ‘unFISH-able’. The most recent reports, however, present binding of MCPIP1 to *IL-6* mRNA (its prototypical and most described substrate) and degradation of this transcript as events separated in time. MCPIP1 first binds to the transcript and awaits for the interaction with another protein, UPF1, before it can degrade the target mRNA (Mino et al., 2015). This time window makes MCPIP1 and *IL-6* mRNA interaction ‘FISH-able’ (Kochan et al., 2015). Consequently, the data obtained from IF-combined smRNA FISH further reinforces and confirms the involvement of MCPIP1 protein in the regulation of *IER3* mRNA stability.

### MCPIP1 protein interacts with IER3 mRNA *in vivo*

**Fig. 4.**
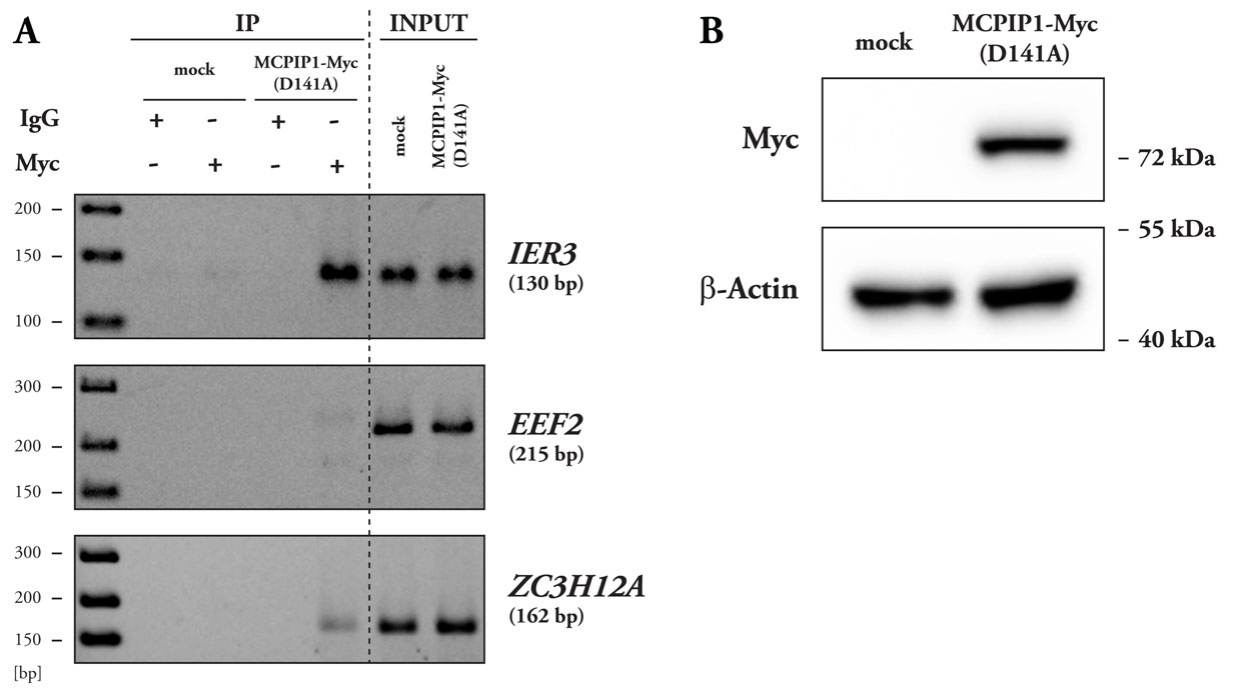
**MCPIP1 interacts with *IER3* mRNA *in vivo*.** (A) HepG2 cells were transfected with empty vector (mock) or with expression vector coding for MCPIP1(D141A)-MycHis and subsequently subjected to RNA immunoprecipitation (RIP) procedure. Representative images of semi-quantitative PCR analysis of RNA eluted after immunoprecipitation are shown. PCR analysis of eluted RNA was performed using primers specific for *IER3* mRNA (upper panel), *EEF2* mRNA (negative control, no specific PCR product obtained, middle panel) or *ZC3H12A* mRNA (positive control, lower panel). (B) Western blot analysis of transfected cells used in RIP showing expression of the MCPIP1-MycHis protein (upper panel). Β-actin was used as a loading control (lower panel). *n*=2.

By the use of smRNA FISH we have proved co-localization of MCPIP1 with *IER3* transcript, however confirmation of their direct interaction required employment of another method. To study this subject we performed RNA immunoprecipitation (RIP). Since good quality of mRNAs is required to detect immunoprecipitated transcripts by RT-PCR we chose a RNase inactive form of MCPIP1, MCPIP1(D141A)-MycHis for these experiments. Obtained results clearly proved that MCPIP1 indeed interacts with *IER3* mRNA *in vivo* (Fig. 4A). In the presence of the antibody against c-myc epitope of MCPIP1(D141A), the *IER3* mRNA was precipitated from cell lysates; simultaneously no precipitation was detected in samples immunoprecipitated with non specific IgG. In contrast, anti-c-Myc antibody was unable to precipitate *EEF2* mRNA above the level observed after precipitation with non specific IgG. Since interaction of MCPIP1 with its own transcript has been reported before, enrichment in *ZC3H12A* mRNA (coding for MCPIP1) served as a positive control of this procedure (Iwasaki et al., 2011; Mino et al., 2015; Mizgalska et al., 2009). For detection of enrichment in endogenous *ZC3H12A* transcript only we used primers targeting the 3′UTR region, these primers did not detect the ectopically expressed MCPIP1(D141A) (lacking 3′UTR). The presence of MCPIP1(D141A)-MycHis protein in the analyzed specimens was verified by western blot analysis revealing a single band of the predicted size of 70 kDa (Fig. 4B).

## DISCUSSION

In recent years the factors controlling mRNA turnover were established as important regulators of eukaryotic gene expression; since then a demand has emerged for the development of reliable methods which would allow simultaneous quantification of transcripts and detection of their binding partners. The most frequently used real-time PCR allows for fast, accurate and reliable quantification of the target transcript in a sample, but it represents the average expression of specific mRNA in a cell population and does not provide information about the proteins associated with the transcript of interest. We have described an efficient method combining IF with smRNA FISH (Kochan et al., 2015), this method allows for simultaneous detection of mRNA and protein quantity and their subcellular distribution in single cells. Using this approach we have shown that both the wild-type and mutant form (inactive NYN/PIN-like domain) of MCPIP1 co-localize with *IER3* mRNA; however, only the presence of wild-type MCPIP1 induces down-regulation of endogenous *IER3* mRNA level. Our results prove applicability of the method that combines IF with smRNA FISH not only for the investigation of the interaction between a protein and a specific RNA but also for the analysis of the impact of this interaction on the mRNA level in single cells. We proved also that the sensitivity of the measurement of mRNA quantity is comparable to qRT-PCR. We identified structures present in *IER3* 3′UTR involved in the MCPIP1-dependent transcript decay. Using the RIP method we have confirmed direct interaction of MCPIP1 and *IER3* mRNA *in vivo*. Our findings are further supported by recently presented results of RNA-immunoprecipitation sequencing (RIP-seq) analysis suggesting *IER3* to be in a pool of mRNAs associated with MCPIP1 protein in resting and IL-1β-stimulated cells (Mino et al., 2015). How does MCPIP1 recognize and target a specific mRNA for degradation? It is a truly intriguing question that still remains to be fully answered. Remarkably, MCPIP1 protein destabilizes target transcripts through their 3′UTRs. It has been reported that MCPIP1 degrades *IL-6*, *IL-1β*, *IL-2* and its own mRNAs through targeting a stem-loop structure located in 3′UTRs of these transcripts (Iwasaki et al., 2011; Li et al., 2012; Matsushita et al., 2009; Mizgalska et al., 2009). Removal of conserved elements containing the stem-loop structures abrogated MCPIP1-mediated destabilization of mRNAs. Although the data obtained so far by different groups suggest clearly that indeed it is the stem-loop structure that drives MCPIP1 targets for degradation, further studies are required to develop a usable methodology allowing for a comparative approach for prediction or identification of regulatory sequences potentially recognized by MCPIP1. Recently, Mino et al. (2015) have made the first step towards identification of the consensus sequence of the stem-loops in MCPIP1 target mRNAs. Using high-throughput sequencing of RNA isolated by crosslinking immunoprecipitation they manage to prepare a preliminary descriptive analysis of MCPIP1-bound stem-loops. Structures with three to seven nucleotide stems and three nucleotide loops were significantly enriched. Additionally, structures with UAU and UGU loops were found to be the most significantly regulated by MCPIP1 overexpression indicating that stem-loop sequences with either A or G at the second residue in the hairpin loop can be important for MCPIP1-dependent suppression. In contrast, ACA, AAA, or UCU stem-loops were found not to be important for MCPIP1 regulation, suggesting that a pyrimidine-purine-pyrimidine (Py-Pu-Py) sequence should be present in the loop. The stem-loop we found in the *IER3* 3′UTR perfectly fits this model. Surprisingly, *IL-6* that is considered the most studied and most strictly regulated MCPIP1 target does not fulfill criteria presented in that work (Mino et al., 2015).

The elements involved in the activation of *IER3* promoter are quite well known. These include consensus sequences for NF-κB, c/EBP, p53, c-Myc, Sp1, p300, Ap1, Ap4, Sox, USF, Elk-1 as well as serum responsive element (SRE), vitamin D responsive element (VDRE), hormone responsive element (HRE) and radiation responsive element (RRE) (Arlt and Schäfer, 2011; Huang et al., 2002; Kochan et al., 2014; Pietzsch et al., 1998). *IER3* belongs to the immediate early genes (IEGs). Induction of these genes does not require *de novo* protein synthesis as it is mediated by pre-existing transcription factors. Since IEGs mRNAs are characterized by quick decay kinetics, two questions arise; first, which proteins are involved in the regulation of these mRNAs half-life; and second, whether the same transcription factors induce expression of IEGs and proteins controlling IEGs transcripts turnover. Until 2013, TTP was the only protein known to be involved in destabilizing *IER3* mRNA; interestingly, *IER3* was described as the TTP top substrate (Lai et al., 2006). Indeed, *IER3* 3′UTR contains several highly conserved AREs crucial for TTP binding and TTP-dependent transcript decay. Linear AREs are, however, only one type of regulatory elements. Recently, it was showed that structured RNA degradation motifs also play significant role in the inflammatory response (Iwasaki et al., 2011; Matsushita et al., 2009). In this paper we have characterized the importance of a stem-loop structure embedded in the non-ARE conserved element of *IER3* 3′UTR resembling structure important for MCPIP1-dependent *IL-6* mRNA destabilization (Matsushita et al., 2009; Paschoud et al., 2006). The herein described regulation of *IER3* mRNA by MCPIP1 and previously described involvement of TTP in this process seem to support the hypothesis that the same transcription factors switch on expression of IEGs and proteins involved in their transcripts degradation. As we have shown previously, IL-1β activates *IER3* promoter via transcription factors NF-κB and Elk-1 (Huang et al., 2002; Kochan et al., 2014). At the same time, IL-1β-induced NF-κB and Elk-1 are also involved in the activation of the genes encoding MCPIP1 and TTP (Florkowska et al., 2012; Kasza et al., 2010; King et al., 2009; Mizgalska et al., 2009; Shah et al., 2016; Skalniak et al., 2009). The cells evolved various mechanisms assuring a short existence of certain proteins that are needed only for a limited time period. It seems that one of them exists in the regulation of transcripts turnover and involves the utilization of the same set of transcription factors for the stimulation of expression of the short-living mRNAs and transcripts of the proteins involved in their degradation.

IER3 was described over two decades ago (Charles et al., 1993; Kondratyev et al., 1996; Schäfer et al., 1996). Despite more than twenty years of elaborate research by several groups, its function persistently remained controversial. IER3 was reported to inhibit cell proliferation in some cells while accelerating cell cycle in others (Charles et al., 1993; Kondratyev et al., 1996; Pietzsch et al., 1997; Schäfer et al., 1999); and exert, depending on the tested system and conditions, pro- or anti-apoptotic effects (Arlt and Schäfer, 2011). IER3 was also reported to negatively regulate NF-kB and inhibit induction of CCL2, IL-6, CXCL1 and IL-1β after TLR2 stimulation (Sina et al., 2010). There are also a growing number of reports suggesting the involvement of IER3 in cancer and inflammatory diseases (Kwon et al., 2013; Zhang et al., 2002). Most recent reports cast new light upon IER3 and reveal its importance in the regulation of inflammatory response. IER3 was described to protect macrophages from LPS-induced cell death (Schott et al., 2014). Macrophages play a central role in the initiation and resolution of inflammation. They respond to exogenous signals such as LPS or cytokines produced at the site of inflammation. These stimulants induce expression of MCPIP1 (Iwasaki et al., 2011; Matsushita et al., 2009). Simultaneously MCPIP1 influences the biology of macrophages. Besides the mentioned down-regulation of pro-inflammatory cytokines synthesis and modulation of cell signaling by deubiquitination of TRAF proteins (Liang et al., 2010), MCPIP1 inhibits polarization of M1 macrophages (Kapoor et al., 2015). Moreover, overexpression of MCPIP sensitizes macrophages to apoptosis under stress (Qi et al., 2011; Wang et al., 2016); thus our finding that MCPIP1 controls *IER3* mRNA turnover could explain the mechanisms of its proapoptotic activity although the final confirmation of this hypothesis requires experimental verification in macrophages.

This study, by presenting the *IER3* mRNA to be a novel MCPIP1 target broadens the knowledge about proteins involved in the transcript turnover. Due to the fact that MCPIP1 plays a key role in controlling inflammatory response, understanding the intricacies of MCPIP1-mediated mRNA degradation paves the way to identifying potential targets for the development of anti-inflammatory drugs.

## MATERIALS AND METHODS

### Cell culture

Human hepatocellular carcinoma cells (HepG2) obtained from American Type Culture Collection (ATCC HB-8065) were cultured in Dulbecco's Modified Eagle's Medium (DMEM) with 1.0 g/l D-glucose (Lonza, Verviers, Belgium) supplemented with 2 mM L-glutamine (Sigma-Aldrich, St. Louis, MO, USA) and 10% (v/v) fetal bovine serum (heat inactivated, South America origin, Biowest, Nuaillé, France). Cells were maintained at 37°C in humidified atmosphere with 5% CO_2_ (Heraeus, Thermo Electron LED GmbH, Langenselbold, Germany). If applied, cells were stimulated with recombinant human interleukin 1β (IL-1β, PromoKine, Heidelberg, Germany) at the concentration of 10 ng/ml. For imaging experiments cells were plated on glass coverslips (15 mm×15 mm, Waldemar Knittel Glasbearbeitungs, Braunschweig, Germany) in 12-well culture plates at the density of 60,000 cells/well in 1 ml of medium 24 h before procedures. For luciferase reporter gene assay experiments cells were plated in 24-well plates at the density of 75,000 cells/well in 0.5 ml of medium 24 h before transfection. For RNA immunoprecipitation cells were plated in 100 mm culture dishes in 15 ml of medium 24 h before transfection. All cell culture plasticware was purchased from BD Falcon (Corning Incorporated, Corning, NY, USA). Cells were counted using TC20 automated cell counter (Bio-Rad Laboratories, Hercules, CA, USA). Cells were cultured without any antibiotics and routinely tested for mycoplasma contamination using PCR (primers sequences are listed in Table S1) or MycoAlert Mycoplasma Detection Kit (Lonza).

### Preparation of plasmid constructs

Human *IER3* 3′UTR was cloned from human cDNA mix and inserted into pmirGLO vector (Promega Corporation, Madison, WI, USA) producing pLuc-IER3-3′UTR. Following primers were used: IER3-3′UTRfor and IER3-3′UTRrev (Table S2). The truncated variant of *IER3* 3′UTR (without non-ARE conserved element containing structurally conserved stem-loop), referred to as pLuc-IER3-3′UTRΔCE, was generated using pLuc-IER3-3′UTR plasmid as a PCR template and the following primers: IER3-3′UTRfor and IER3-3′UTRdCErev. A SnapGene DNA sequence file presenting these two constructs in detail is added as supplementary data file. Preparation of pLuc-IL6-3′UTR, pLuc-IL6-3′UTRΔCE and human wild-type (WT) MCPIP1-MycHis expression vector was described previously (Kochan et al., 2015). The expression vector coding for RNase-inactive mutant of MCPIP1, referred to as MCPIP1(D141A)-MycHis was obtained with QuikChange II XL site directed mutagenesis kit (Stratagene, La Jolla, CA, USA) using plasmid coding for WT form as a template and the following primers: M1-D141Asense and M1-D141Aantisense (Table S3). The enhanced green fluorescent protein (EGFP) coding sequence was cloned from pIRES2-EGFP vector (Clontech, Mountain View, CA, USA) and inserted into pcDNA3.1/MycHisA vector (Invitrogen, Carlsbad, CA, USA) producing EGFP-MycHis expression vector using the following primers: EGFPmycFOR and EGFPmycREV (Table S2). All restriction endonucleases, T4 DNA ligase and Calf Intestinal Alkaline Phosphatase (CIP) were obtained from New England Biolabs, Ipswich, MA, USA. In PCR-based cloning, *Pfu*Ultra II Fusion HS DNA Polymerase (Agilent Technologies, Santa Clara, CA, USA) and Veriti 96-Well Fast Thermal Cycler (Applied Biosystems, Foster City, CA, USA) were used. Primers used in cloning (listed in Tables S2 and S3) were obtained from Genomed, Warszawa, Poland. All obtained constructs were verified by Sanger sequencing (Genomed). For expression plasmids, recombinant protein expression was confirmed by western blotting.

### Transfections

HepG2 cells were transfected using Lipofectamine 2000 reagent (Invitrogen) according to the manufacturers’ instructions, with plasmid DNA propagated in One Shot TOP10 Chemically Competent *E. coli* (Invitrogen), purified using a Syngen Plasmid MIDI Kit (Syngen, Wrocław, Poland) and resuspended in water free of nucleases and proteases (Sigma-Aldrich). Transfection medium was changed after 4 h.

### Luciferase reporter gene assay

A total amount of 800 ng of plasmid DNA per well was used containing 100 ng of pmirGLO dual luciferase expression vector (pLUC-IER3-3′UTR, pLuc-IER3-3′UTRΔCE, pLUC-IL-6-3′UTR, pLuc-IL-6-3′UTRΔCE or empty pmirGLO) and 12.5 ng of MCPIP1-MycHis expression vector. The amount of DNA per well was leveled to 800 ng with pcDNA3.1/MycHisA 24 h after transfection cells were lysed and assayed for luciferase activity using Dual-Luciferase Reporter Assay System (Promega Corporation) according to the manufacturers’ instructions. *Renilla* luciferase was used as internal control.

### Quantitative real-time PCR (qRT-PCR) experiments

Isolation of total RNA, reverse transcription and qRT-PCR were done similarly to procedures described previously (Kochan et al., 2014). Concisely, total RNA was isolated according to the protocol developed by Chomczynski and Sacchi (2006). Following isolation, RNA quality was assessed using agarose gel electrophoresis under denaturing conditions. RNA quantity was measured using a NanoDrop spectrophotometer (Thermo Fisher Scientific, Wilmington, DE, USA), and equal amounts of 1 μg were used in the reverse transcriptase reaction to generate cDNA (M-MLV Reverse Transcriptase; Promega Corporation). For qRT-PCR, DNA fragments were amplified using SYBR qRT-PCR Kit (A&A Biotechnology, Gdynia, Poland), and primers specific to the transcripts being assessed (*IER3* and *EEF2* as reference gene). Fluorescence was measured using the StepOnePlus Real-Time PCR System (Applied Biosystems, Waltham, MA, USA). *IER3* mRNA level in each sample was analyzed in duplicates, and the expression level was normalized to the level of *EEF2* mRNA. The ΔΔC_q_ method was used to calculate the final results (Livak and Schmittgen, 2001). Primers used in qRT-PCR (listed in Table S4) were obtained from Genomed. Presence of single qRT-PCR products was confirmed by agarose gel electrophoresis and melting curve analysis.

### Single- molecule RNA fluorescence *in situ* hybridization (smRNA FISH)

For smRNA FISH experiments performed exclusively, the procedure was carried out according to the probe blend suppliers’ instructions (LGC Biosearch Technologies, Inc., Petaluma, CA, USA). Custom-designed probe blend labeled with Quasar 570 dye targeting *IER3* mRNA was used at the concentration of 250 nM. Sequences of custom probe set are listed in Table S5. All hybridizations were carried out overnight in the dark at 37°C in a humidifying chamber. Imaging details are presented in the next section.

### IF-combined smRNA FISH

Simultaneous protein and mRNA detection using immunofluorescence-combined single-molecule RNA fluorescence *in situ* hybridization was performed as described previously (Kochan et al., 2015). Briefly, HepG2 cells were plated, as described above, on glass coverslips in 12-well culture plates at the density of 60,000 cells/well in 1 ml of medium one day before transfection. 24 h after transfection cells were fixed for 10 min in 4% methanol-free formaldehyde (Thermo Scientific, Rockford, IL, USA) in 1× RNase-free PBS at room temperature. Next, specimens were blocked and permeabilized for 60 min at room temperature in blocking buffer (1× RNase-free PBS, 1% acetylated BSA, 0.3% Triton X-100, 2 mM vanadyl ribonucleoside complexes). Blocked specimens were incubated with antibodies diluted in blocking buffer. MCPIP1-MycHis and MCPIP1(D141A)-MycHis proteins were stained with anti-c-Myc antibody (mouse, clone 9E10, MA1–980; 1:200; Thermo Scientific) and Alexa Fluor 488-conjugated donkey anti-mouse antibody (1:150, 715-546-150, Jackson ImmunoResearch, West Grove, PA, USA). Incubations with primary antibodies were carried out overnight in the dark at 4°C and with secondary antibodies for 90 min in the dark at room temperature, in a humidifying chamber. After post-fixation (10 min in 4% methanol-free formaldehyde in 1× RNase-free PBS at room temperature), smRNA FISH procedure was performed described above. Finally all samples were mounted onto slides in Vectashield Mounting Medium with DAPI (Vector Laboratories, Burlingame, CA, USA), sealed with nail polish and imaged using Leica DMI6000B (AF7000 version) inverted widefield fluorescence microscope (Leica Microsystems, Wetzlar, Germany). All images were recorded using a high numerical aperture 100× oil immersion objective [HCX PL APO 100.0×1.47 oil; Leica Microsystems; immersion oil, Nikon 50 Type A, n_D_ (refractive index)=1.515 (at 23°C); Nikon Instruments Europe BV, Amsterdam, The Netherlands] using a 14-bit Hamamatsu 9100–02 EM-CCD High Speed Set cooled CCD camera (Hamamatsu Photonics, Hamamatsu, Japan) with Leica LAS X image acquisition software. The following filter sets (Leica Microsystems) were used: A4 for detection of DAPI, GFP-T ET for detection of Alexa Fluor 488 Dye and GFP and Rhod ET for detection of Quasar 570 Dye. After deconvolution from about 60 *z*-sections with 0.15-0.20 μm spacing, images were analyzed by local background subtraction and thresholding using Huygens Software (Scientific Volume Imaging, SVI, Hilversum, The Netherlands). To quantify co-localization, both imaging channels were correlated pixel-wise and the Pearson coefficient was calculated (using Huygens Colocalization Analyzer and Surface Renderer, SVI). Final image adjustments were performed using ImageJ 1.48v (National Institutes of Health, Bethesda, MD, USA), Adobe Photoshop CS4 Extended Version 11.0.2, and Adobe Illustrator CS4 Version 14.0.0 (Adobe Systems, San Jose, CA, USA).

### RNA immunoprecipitation (RIP)

To perform immunoprecipitation of mRNA bound to MCPIP1(D141A)-MycHis protein, 2×10^6^ HepG2 cells were plated in 100 mm cell culture dishes in 15 ml of medium. The following day cells were transfected with plasmid coding for MCPIP1(D141A)-MycHis or empty vector (pcDNA3.1/MycHisA) using Lipofectamine 2000 reagent (Invitrogen) according to the manufacturers’ instructions and 4 h after transfection medium was changed. After 24 h transfection cells were washed twice with 1× PBS, 1 ml of 1× PBS per dish was added and RNA-protein complexes were crosslinked by UV-irradiation of the cells (300 mJ/cm^2^) at 254 nm (Hoefer UVC 500 Ultraviolet Crosslinker, Hoefer, San Francisco, CA, USA). Next, cells were dislodged in 1× PBS using a cell scraper, transferred to microfuge tubes and pelleted (300×***g***, 5 min, at 4°C). Supernatants were removed and pellets were resuspended in 300 µl of RIP sample buffer (10 mM HEPES pH 7.5, 10 mM KCl, 0.1 mM EDTA, 0.1 mM EGTA, 1 mM DTT) supplemented with 0.2 mM PMSF (Sigma-Aldrich), 1× cOmplete protease inhibitors (Roche Diagnostics GmbH, Mannheim, Germany) and 1 unit/µl of murine RNase inhibitor (New England Biolabs). After 10 min of incubation on ice, NP-40 (BioShop, Burlington, Ontario, Canada) to a final concentration of 0.8% was added, samples were vortexed for 10 s and centrifuged at 13,000×***g*** for 3 min at 4°C. Obtained supernatants were used in the following procedure. 100 µl of each sample (one third) were used for immunoprecipitation. Before addition of antibodies each sample was diluted five times in RIP dilution buffer (16.7 mM HEPES pH 7.5, 167 mM NaCl, 1.2 mM EDTA, 1.1% Triton X-100, supplemented with inhibitors as described above). mRNA-protein complexes were immunoprecipitated overnight at 4°C with gentle rotation using 2 µg of anti-c-Myc antibody (mouse, clone 9E10, MA1–980; Thermo Scientific). 2 µg of normal mouse IgG (Thermo Scientific) were used as a control of specificity. The following day, the complexes were recovered using Dynabeads Protein G (Novex, Thermo Scientific) pre-washed with RIP dilution buffer. After 90 min of incubation at 4°C, the beads were washed once with 1 ml of each of the following buffers in the presented order: RIP low salt wash buffer (20 mM HEPES pH 7.5, 150 mM NaCl, 2 mM EDTA, 1% Triton X-100, 0.25% sodium deoxycholate), RIP high salt wash buffer 1 (20 mM HEPES pH 7.5, 500 mM NaCl, 2 mM EDTA, 1% Triton X-100, 0.25% sodium deoxycholate) and RIP high salt wash buffer 2 (20 mM HEPES pH 7.5, 500 mM NaCl, 2 mM EDTA, 1% Triton X-100, 0.5% sodium deoxycholate, 0.01% SDS). Each 5 min wash was performed at room temperature and buffers were removed from the beads suspension on a magnet (Dynal MPC-S, Thermo Scientific). Finally beads were resuspended in Proteinase K digestion buffer (10 mM Tris-HCl pH 8.0, 100 mM NaCl, 1 mM EDTA, 0.5% SDS) and treated with 200 µg/ml Proteinase K (New England Biolabs) for 1 h at 50°C. Inputs were similarly treated with Proteinase K. Following digestion, RNA was isolated by phenol-chloroform extraction and isopropanol precipitated (overnight at −20°C) with Pellet Paint Co-Precipitant (Novagen, Merck Millipore, Darmstadt, Germany). The RNA pellets were resuspended in nuclease-free water (Sigma-Aldrich) and reverse transcribed to generate cDNA (M-MLV Reverse Transcriptase; Promega Corporation) – for input samples 1/7th of the obtained RNA was used – and analyzed by semi-quantitative PCR. DNA fragments were amplified using StartWarm PCR Master Mix (A&A Biotechnology, Gdynia, Poland), and primers specific to analyzed transcripts of *IER3*, with *EEF2* as negative control and *ZC3H12A* as positive control. PCR products were separated in 3% agarose gel (Serva 3:1 agarose, Serva, Heidelberg, Germany) in 1× TBE buffer and visualized using standard ethidium bromide staining protocol. Amount of input RNA content was verified using qRT-PCR and similar C_q_ for each sample were recorded. Primers used in RIP procedure (listed in Table S6) were obtained from Genomed.

### Western blot analysis

Protein extracts [10 μl of RIP cell lysates, treated with RNase A (10 µg per sample, Sigma-Aldrich) for 60 min at 37°C] were separated by SDS-PAGE and wet-transferred onto an Immobilon-P PVDF membrane (Millipore Corporation, Billerica, MA, USA). The membranes were blocked for 1 h at room temperature in 5% w/v nonfat dry milk (BioShop) in TTBS (20 mM Tris, 150 mM NaCl, 0.1% Tween-20). After blocking, membranes were incubated overnight with gentle agitation at 4°C with the following primary antibodies: anti-c-Myc (mouse, clone 9E10, MA1–980; 1:16,000; Thermo Scientific) or anti-β-actin (rabbit, clone 13E5, #4970, 1:5000; Cell Signaling Technology, Danvers, MA, USA), washed three times for 5 min at room temperature each with 25 ml TTBS and incubated with HRP-conjugated secondary antibodies (anti-mouse or anti-rabbit; 1:5000; Cell Signaling Technology) for 1 h at room temperature. All antibodies were diluted in blocking buffer. After incubation with secondary antibodies, membranes were washed three times for 5 min at room temperature each with 25 ml TTBS, and the chemiluminescence (Clarity Western ECL Blotting Substrate; Bio-Rad Laboratories) was detected using Fusion-Fx system (Vilber Lourmat, Eberhardzell, Germany).

### Data sources for sequence alignments

For sequence and structure conservation analysis of *IER3* 3′UTRs, the following GenBank mRNA 3′UTR sequences were retrieved from the National Center for Biotechnology Information (NCBI) database: *Homo sapiens* (human, NCBI Reference Sequence: NM_003897.3), *Chlorocebus sabaeus* (green monkey, XM_007973201.1), *Gorilla gorilla* (western gorilla, XM_004043597.1), *Oryctolagus cuniculus* (rabbit, XM_002714361.2), *Pan troglodytes* (chimpanzee, NC_006473.3), *Bos taurus* (cattle, NM_001075202.2), *Callithrix jacchus* (white-tufted-ear marmoset, XM_002746312.2), *Capra hircus* (goat, XM_005696631.2), *Equus caballus* (horse, XM_001491016.4), *Felis catus* (domestic cat, XM_003985890.3), *Mus musculus* (house mouse, NM_133662.2), *Ovis aries* (sheep, XM_004018996.3), *Pongo abelii* (Sumatran orangutan, XM_002816638.2), *Rattus norvegicus* (Norway rat, NM_212505.2), *Sus scrofa* (pig, XM_001927551.3). All sequences were downloaded from NCBI on March 1, 2016. Clustal Omega, mfold and LocARNA web servers were used with the default settings.

### Statistical analysis and graphs

All graphs and statistical analyses were done using GraphPad Prism (version 6.07, GraphPad Software, Inc., La Jolla, CA, USA). Two-way ANOVA with post-hoc Bonferroni test (Fig. 1F), two-tailed *t*-test (Fig. 2B,D) or one-way ANOVA with post-hoc Bonferroni test (Fig. 3B) were used. Details of statistical analyses, calculated significances and plotted values for each graph are presented in figure legends.
